# Pharmacogenomics of Medications Commonly Used in the Intensive Care Unit

**DOI:** 10.3389/fphar.2018.01436

**Published:** 2018-12-04

**Authors:** Shuqin Zhou, Debra J. Skaar, Pamala A. Jacobson, R. Stephanie Huang

**Affiliations:** ^1^Department of Emergency and Critical Care Medicine, Shanghai Tenth People's Hospital, Tongji University, Shanghai, China; ^2^Department of Experimental and Clinical Pharmacology, College of Pharmacy, University of Minnesota, Minneapolis, MN, United States

**Keywords:** pharmacogenomics, personalized medicine, pharmacogenetics, intensive care unit, adverse drug reaction

## Abstract

In the intensive care unit (ICU) setting, where highly variable and insufficient drug efficacies, as well as frequent and unpredictable adverse drug reactions (ADRs) occur, pharmacogenomics (PGx) offers an opportunity to improve health outcomes. However, PGx has not been fully evaluated in the ICU, partly due to lack of knowledge of how genetic markers may affect drug therapy. To fill in this gap, we conducted a review to summarize the PGx information for the medications commonly encountered in the ICU.

## Introduction

Patients in the intensive care unit (ICU) often have multiple serious medical conditions. The critical and complex medical conditions necessitate patients' admission to ICU. Pharmacotherapy is a key component of critical care medicine. ICU patients are vulnerable and cannot afford the comorbidities due to a drug treatment failure. The challenge of pharmacotherapy in the ICU is that patients may concurrently receive the medical treatment of multiple sedative-analgesics, antibiotics, antifungal drugs, anticoagulants, antiarrhythmics, and sometimes paralytics as well as medications to inhibit gastric acid secretion. Critical illness combined with preexisting chronic diseases often results in hepatic or renal insufficiency and, as a consequence, alterations in patient body's disposition and response to drugs. Therefore, the choice of right medication at the right dose is challenging, but essential, to avoid severe and unexpected toxicities and to ensure therapeutic efficacy. Traditional ICU decision-making strategies for treatments depend on the experience of critical care providers, individual patient characteristics and environmental influences. Despite of these efforts, treatment effectiveness varies among ICU patients. The incidence of adverse drug reactions (ADRs) in ICU is much higher than that in other clinical environments (Grenouillet-Delacre et al., 2007; Joshua et al., 2009). Additionally, the expense of pharmaceuticals in the critical care environment is huge. Not only intensive care clinicians but also the pharmaceutical industry are seeking predictors to avoid the risk of ADRs, predict response and reduce the costs of pharmaceuticals in the ICU (Empey, 2010; Patterson et al., 2011). With the emergence and development of human genome sequencing technologies and high throughput genetic analysis, it has been established that genetic variants play a role in an individual's response to drugs. Pharmacogenomics (PGx), a relatively young research field, which aims at deciphering the relationship between individuals' genetic makeup and medication responses, has yield promising results by allowing the prediction of drug responses and the prevention of ADRs, using patient genetic information (Carr and Pirmohamed, 2018). Taking together, incorporating PGx into clinical decision-making in the ICU may improve the patient treatment outcomes significantly (Su et al., 2014; MacKenzie and Hall, 2017). Indeed, PGx has been investigated in a variety of therapeutic areas: cancer, cardiovascular disease, depression, and human immunodeficiency virus infection as well as rheumatologic disorders. The majority of the drugs that are labeled with PGx information by the U.S. Food and Drug Administration (FDA) are used in oncology, cardiology or psychiatry. Considerable PGx research has been conducted in the anesthesia literature (Behrooz, 2015). This data suggests that specific pharmacokinetic genotype screening could personalize the treatment of analgesics used in the ICU (Landau et al., 2008). The potential utility of applying the PGx approach to guide the selection and dosage of drugs for patients who are acutely ill has a promising future.

However, the PGx research has mainly focused on subacute or chronic illnesses. The ICU setting has not been chosen for conducting PGx research or implementation due to a number of challenges, for example, the lack of knowledge on how PGx markers may affect drug therapy in the ICU setting. The complex medical problems seen in the ICU require well-designed studies with adequate power to account for multiple confounding factors. To facilitate the evaluation of PGx in the ICU and to fill in the knowledge gap, we conducted a review of the PGx information for medications that are commonly used in the ICU setting. The choice of drugs to be evaluated was based on two criterion: first, the medications are frequently utilized in the ICU. Secondly, there needs to be substantial literature support of PGx information for these medications, specifically, existence of guidelines, and/or FDA drug labels containing PGx information. Note that the requirement of the second criteria rules out some very commonly used ICU medications, for example, insulin, and hydrocortisone. In addition, we propose several practical steps to encourage PGx research in the ICU.

## Pharmacogenomics of Commonly Used ICU Medications

### Genetic Terms and Definitions

The Clinical Pharmacogenetics Implementation Consortium (CPIC) is an international consortium with the goal to translate laboratory genetic test information into actionable prescribing suggestions for related medications (Caudle et al., 2014a). The Dutch Pharmacogenetics Working Group (DPWG) was established to develop pharmacogenetics-based therapeutic (dose) recommendations (Swen et al., 2011). These guidelines provide information on the strongest evidence to-date between genetic information and medication usage. They meant to provide support for the usage of PGx information in a specific disease setting. However, none of these guidelines should be viewed as a mandate for a genetic test prior to prescribing medications. CPIC has created standardized terms to be used in their guidelines and in the larger pharmacogenetics community. A variant form of genes is an allele. In star allele nomenclature, alleles are identified through the means of numbers and letters, separated from the gene name by a star. For example: CYP3A5^*^3 is a typical nonfunctional variant of *CYP3A5*. CYP3A5^*^3 identifies the genetic variant at the genomic position 22,893 on chromosome 7 (7q21.1). A variant from A to G creates aberrant splicing of *CYP3A5* mRNA, resulting in nonfunctional protein. CYP3A5^*^3 has a dbSNP number: rs776746. The star allele nomenclature is thought to be faster and easier for non-specialized professionals in identifying important PGx alleles. The star allele nomenclature has been firstly used to identify alleles within the cytochrome P450 (CYP) gene family. The Pharmacogene Variation (PharmVar, at www.PharmVar.org, former at www.cypalleles.ki.se) database is a critical resource for the PGx research. This site provides allele nomenclatures for a collection of very important pharmacogenes (Gaedigk et al., 2018). From that, it spread to all genes that have been studied in PGx (Kalman et al., 2016). The variants of the CYP450 genes (e.g., *CYP2D6, CYP2C19*, and *CYP2C9* genes) were discovered on the basis of the metabolization status variants in medication treated individuals. Caudle et al. worked on the standardization of the PGx terms to capture the phenotypic differences in these gene activities. The phenotypic definitions of ultrarapid metabolizer (UM), rapid metabolizer (RM), extensive metabolizer (EM), intermediate metabolizer (IM), and poor metabolizer (PM) are basing on the amounts and categories of function alleles (increased, normal or decreased). Normal metabolizer (NM) has taken place of EM in CPIC guidelines published after 2017 (Bank et al., 2018). Therefore, for this review as summary sentences, we will use UM, RM, EM/NM, IM, and PM to depict phenotypes of different CYP450 enzymes. The exact definition of genetic effect grouping for each gene can be found in the CPIC term standardization project (Caudle et al., 2017).

### Commonly Used ICU Drugs

To summarize the PGx information for the commonly encountered drugs in ICU, we grouped the drugs into five categories: analgesics and sedatives, antifungal drugs, cardiovascular drugs, gastroenterological drugs, and anticonvulsant drugs. Only those genes that have been confirmed to relate to more than one commonly prescribed ICU drugs are listed in Table 1. According to the CPIC guidelines, we develop clinically actionable flow charts (Figure 1) to facility the adaptation of genomic information in clinical decision-making (Caudle et al., 2016).

**Table 1:** Summary of key pharmaco-genes for commonly prescribed ICU medications.

** 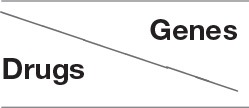 **	***CYP2D6***	***CYP2C19***	***CYP3A4/5***	***CYP2C9***	***HLA-B***
Tramadol ^‡^	**+**			
Codeine ^*^^†^^‡^	**+**			
Oxycodone ^‡^	**+**			
Haloperidol ^‡^	**+**			
Carvedilol ^*^^‡^	**+**			
Metoprolol ^*^^‡^	**+**			
Propafenone ^*^^‡^	**+**			
Ondansetron ^*^^†^	**+**			
Voriconazole ^†^^‡^		**+**		
Clopidogrel ^*^^†^^‡^		**+**		
PPIs^*#*^^*‡*^		**+**		
Diazepam ^*^		**+**	**+**	
Midazolam			**+**	
Fentanyl			**+**	
Warfarin ^†^				**+**
Phenytoin ^*^^†^^‡^				**+**	**+**
Carbamazepine ^*^^†^					**+**

To summarize the PGx information for the drugs commonly encountered in the ICU. Only those genes that have been confirmed to relate to more than one commonly prescribed ICU medications are listed. + Literatures, FDA or/and guidelines (CPIC, DPWG) supported;

* Drug has FDA approved label with PGx biomarkers;

† PGx-based drug dosing guidelines published by the Clinical Pharmacogenetics Implementation Consortium (CPIC);

‡ PGx-based drug dosing guidelines published by the Royal Dutch Association for the Advancement of Pharmacy-Pharmacogenetics Working Group (DPWG);

# *PPIs, Proton Pump Inhibitors (Omeprazole, Esomeprazole, Lansoprazole, Pantoprazole)*.

**Figure 1:**
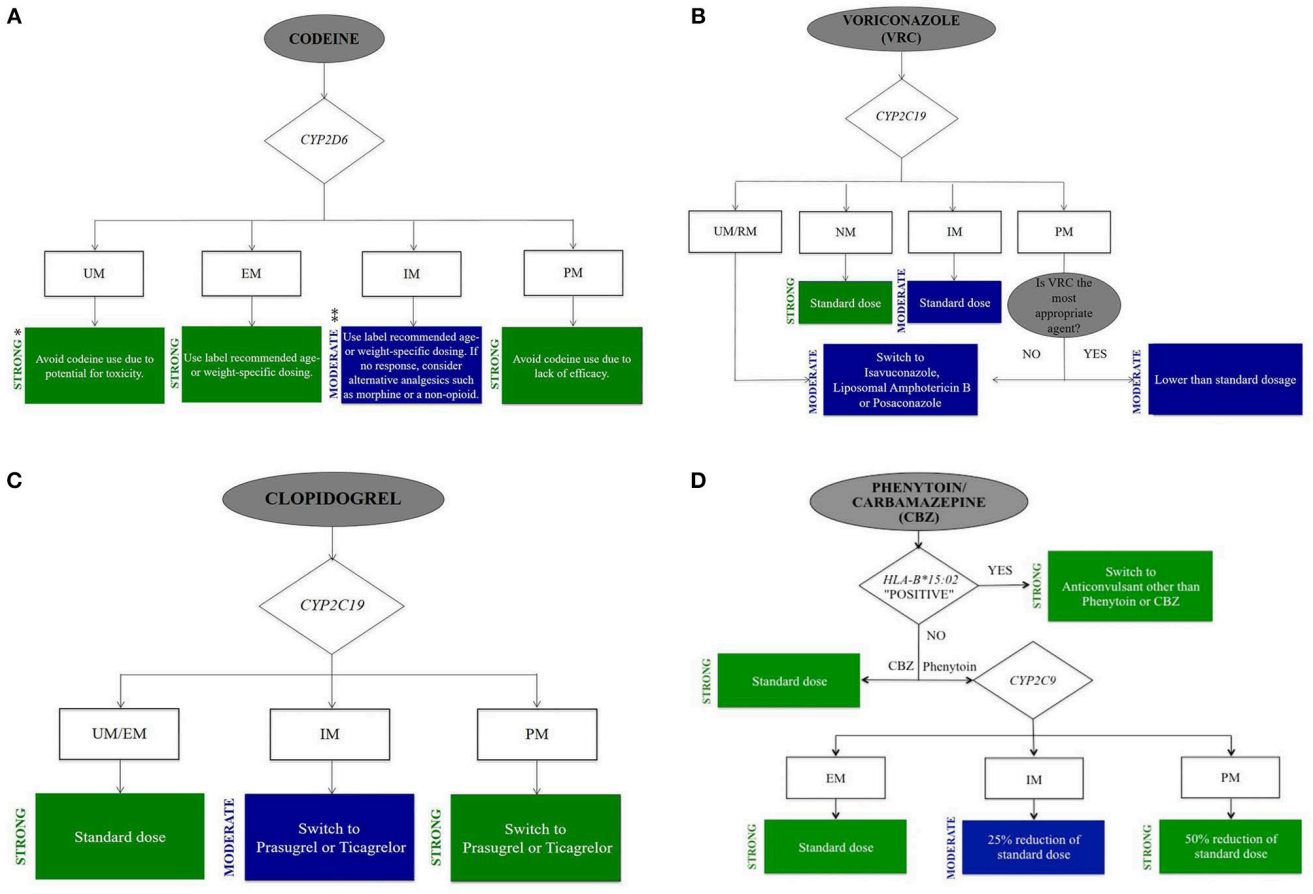
Clinical actionable flow chart of drugs commonly used in ICU. **(A)** Codeine. **(B)** Voriconazole. **(C)** Clopidogrel. **(D)** Phenytoin/Carbamazepine. Develop clinical actionable flow chart based on CPIC guidelines to facility the adaptation of genomic information in clinical decision-making in ICU. Evidence-based recommendation taken from the online CPIC: Strength of Recommendations, https://cpicpgx.org/strength-of-recommendations/. * “Strong” means “the evidence is high quality and the desirable effects clearly outweigh the undesirable effects.” ** “Moderate” means “there is a close or uncertain balance as to whether the evidence is high quality and the desirable clearly outweigh the undesirable effects.”

#### Analgesics and Sedatives

##### Opioid analgesics

*Morphine*. Because of the global accessibility and extensive clinical experience, morphine has become one of the most widely used opioids. High interindividual differences in analgesia generated by morphine is well-known and a clinically important challenge. There is increasing evidence that the reaction to morphine can be modulated by pharmacokinetic (PK) and pharmacodynamic (PD) related allelic genetic variants.

Variants affecting morphine metabolism

Two major metabolites of morphine are morphine-6-glucuronide (M6G) and morphine-3-glucuronide (M3G). The uridine diphosphate glucuronosyltransferase (UGT) 2B7 is the metabolizing enzyme (Smith, 2009). Among patients receiving morphine therapy with *UGT2B7* variants at position −79, the ratios of M6G/morphine and M3G/morphine reduced compared with non-carriers of this polymorphic change (De Gregori et al., 2016). A recent randomized crossover trial in healthy individuals showed no association between different *UGT2B7* variants and morphine PK (Nielsen et al., 2017). Research investigating the effects of *UGT2B7* genotypes on morphine glucuronidation activity was limited and the results were inconsistent across different study approaches. It has still not yet been completely clear for what affect the genetic variability has on morphine in the acute pain setting. Thus, it is still unclear for *UGT2B7* variants' clinical impact and further investigation is needed.

Variants affecting morphine PD

The analgesic effect of morphine is mainly exerted by binding to μ-opioid receptor in the central nervous system. The μ-opioid receptor is encoded by the *OPRM1* gene and the A118G nucleotide substitution is the most commonly researched variant. Studies conducted on cancer and postoperative patients treated with morphine showed that individuals who carrying the homozygote G allele had higher pain scores and required higher doses (Nielsen et al., 2015). Huang et al. conducted a meta-analysis comprising more than 4,000 individuals and showed that those who were GG homozygous required 40% higher postoperative morphine. This was mainly because over 40% of Asian population had the G allele compared to only 15% of the European population did (Hwang et al., 2014). COMT enzyme modulates the neurotransmission of norepinephrine and dopamine. Variability in *COMT* has been proved to affect the efficacy of morphine. For patients who carried the G/G genotype of *COMT* (rs4680; p.Val^158^Met), the morphine dose required on day 1 was higher in comparison with those who carried the A/A and A/G genotypes of *COMT* (Matsuoka et al., 2017). In clinical practice, variability in *COMT* was related to morphine's central ADRs such as confusion and hallucinations (Ross et al., 2008). A recent postoperative study showed that compared with single genetic variants, combinations of *COMT* and *OPRM1* variants provided a better explanation of the variability in the metabolism of morphine (De Gregori et al., 2016). Hence, several genetic variants may affect protein function synergistically and multi-gene models will be needed in practice.

Variants affecting morphine transporters

P-gp is an efflux transporter for morphine that is encoded by the *ABCB1* gene. An observational study in cancer patients has found that there is an correlation among *ABCB1* variants and altered analgesic responses as well as dose requirements (Lötsch et al., 2009). Campa et al. showed that those with C3435T variant had a substantially increased morphine analgesic effect in pain patients (Campa et al., 2008). In 263 pediatric surgical patients undergoing tonsillectomy, there were higher risks of opioid-related respiratory depression and prolonged hospital stays in children with GG and GA genotypes (Sadhasivam et al., 2015).

*Fentanyl*. Fentanyl, a first-line analgesic in the ICU, is a synthetic opioid analgesic commonly prescribed for its ability to provide both analgesia and sedation. It is the preference analgesic for patients with kidney failure. In adult critically ill patients, variability in response to fentanyl, including PK and PD, was large (Choi et al., 2016).

Variants affecting fentanyl PK

Fentanyl is N-dealkylated by CYP3A4 and CYP3A5 enzymes into the inactive norfentanyl (Feierman and Lasker, 1996). Variations in therapeutic efficacy of fentanyl can be partly explained by PK factors. However, mixed results were discovered for the CYP3A5^*^3 variants and its effects on PK. Homozygous CYP3A5^*^3 gave rise to decreased metabolism of fentanyl, so the toxicity might be identified by genotyping CYP3A5^*^3 variant (Jin et al., 2005). Barratt et al. however, pointed out that only < 2% of the metabolism of fentanyl was associated with the CYP3A4^*^22 and CYP3A5^*^3 variants and the plasma concentrations of fentanyl were not affected (Barratt et al., 2014). Jin M et al. indicated that individuals with homozygous CYP3A5^*^3 have reduced fentanyl clearance. The fentanyl toxicity was likely to reduce by identifying *CYP3A4/5* PM alleles (Ahmed et al., 2005).

Variants affecting fentanyl PD

The effect of fentanyl is exerted through the binding with μ-opioid receptor. Variability of pain sensitivity may be affected by genetic variants in opioid receptors (OPRK1, OPRM1, and OPRD1). Within a total of 189 patients receiving midazolam and fentanyl postoperatively, the reduced analgesic effect of fentanyl and shortened time period to recover consciousness were observed to be associated with the *OPRM1* 118A>G variant (Wu et al., 2009). Due to decreased expression and activity of p-glycoprotein, *ABCB1* variant rs1045642 AA was found associated to fentanyl dosage of pediatric critical ill patients (Horvat et al., 2017). However, Klepstad et al. studied 695 cancer patients with the treatment of fentanyl. There was no association observed between opioid requirements and the genetic variants of *OPRM1* and *ABCB1* (Klepstad et al., 2011).

*Tramadol*. Tramadol is an opioid analgesic and a 5-HT transporter inhibitor. The O-demethylated metabolite of tramadol is a potent μ-opioid agonist (Barann et al., 2015). If patients are overdosed on tramadol, they will face a major risk of seizures and respiratory depression. Stamer et al. found respiratory depression in one patient treated with tramadol. Genotyping revealed this patient to be a CYP2D6 UM, which resulted in the increased O-desmethyltramadol (Stamer et al., 2008). The generation of O-desmethyltramadol, active metabolite of tramadol, significantly contributes to the drug's activity (Srinivas, 2015). Tramadol PK are characterized by large interindividual variability, which is partially attributed to variants in *CYP2D6*. CYP2D6 PMs have lower median plasma area under the curve (AUC) of O-desmethyltramadol in comparison to CYP2D6 NMs (Stamer et al., 2007). As compared with CYP2D6 NMs, PMs more often had reduced analgesia in response to tramadol (Kirchheiner et al., 2008; Xu et al., 2014). The guideline from DPWG recommends PMs to “receive an alternative drug (not oxycodone or codeine) or monitor the clinical symptom of pain relief.” For IMs, the DPWG recommends to “monitor closely the reduced tramadol efficacy and to consider raising the dose of tramadol.” If the reaction is still insufficient, it is recommended to “use an alternative drug (not oxycodone or codeine).” For CYP2D6 UMs, the DPWG recommends, “an alternative drug for tramadol (not oxycodone or codeine) or to decrease the dose 30% and monitor closely for potential ADRs” (Stamer et al., 2007).

*Codeine*. Codeine is an opioid analgesic to treat mild to moderate pain. A potentially serious ADR of codeine is respiratory depression. Codeine is bioactivated to morphine by the hepatic CYP2D6; thus, codeine's efficacy and safety is dependent on the activity of CYP2D6. PK/PD researchers showed a reduction in morphine concentrations and analgesic effect of codeine in CYP2D6 PMs compared with NMs (Poulsen et al., 1996). In contrast, PK tests of codeine indicated that there were more conversions to morphine within CYP2D6 UMs in comparison with NMs, which leaded to toxic systemic morphine concentrations even when the doses of codeine were low (Kirchheiner et al., 2007). The black box warning of FDA approved drug labeling states that, “respiratory depression and death have occurred in children who received codeine following a tonsillectomy and/or adenoidectomy and who had evidence of being CYP2D6 UMs” (U.S Food and Drug Administration, 2018a). The 2014 CPIC guideline for codeine therapy recommends that for patients who carrying the *CYP2D6* PM or UM alleles, “alternative analgesics for codeine should be used.” For IMs, the CPIC guideline recommends, “specific doses based on age or weight.” Alternative analgesics (morphine or a non-opioid) are recommended to non-responders (Figure 1A) (Crews et al., 2014). DPWG guideline recommends alternative analgesics for PMs, IMs, or UMs (Swen et al., 2011).

*Oxycodone*. Two main metabolites of oxycodone are active oxymorphone and inactive noroxycodone metabolized by CYP2D6 enzyme and CYP3A4 enzyme, respectively. A decrease in the activity of CYP2D6 enzyme reduced the conversion of oxycodone to active oxymorphone (Kummer et al., 2011). The recommendation of the DPWG is to “use an alternative to oxycodone (not codeine or tramadol) in CYP2D6 PMs and IMs or remain constantly alert to deficient relief of pain.” In the case of CYP2D6 UM, “a substitute to oxycodone (not codeine or tramadol) should be used or remain constantly alert to potential ADRs” (de Leon et al., 2003).

##### Sedatives

*Propofol*. Propofol is an commonly used anesthetics and sedatives in the ICU. It is glucuronidated and hydroxylated to its inactive metabolite. CYP2B6 enzyme takes the major responsibility for the individuals' variability in hydroxylation rate (Court et al., 2001). Mourão et al. showed that the *CYP2B6* c.516G>T genetic variant decreased the metabolism of propofol and accounted for ~7% of the propofol dose variation (Mourão et al., 2016). Mastrogianni et al. indicated that the same *CYP2B6* polymorphism was associated with increased propofol concentrations in Greek women (Mastrogianni et al., 2014). The findings of these studies showed that the genetic variations associated with propofol metabolism could improve the prediction of therapeutic response to propofol.

*Midazolam*. Midazolam is commonly used benzodiazepine for sedation in the emergency departments and ICU. Midazolam is mainly metabolized by CYP3A4/3A5 enzyme to the active metabolite, alpha 1-hydroxymidazolam (Spina and Ensom, 2007). Variability in midazolam metabolism may partly due to genetic variants of the *CYP3A* alleles (Lin et al., 2002). Individuals with CYP3A5^*^1/^*^3 had a higher activity of midazolam hydroxylation in comparison with those who carrying CYP3A5^*^3/^*^3 variant (Kuehl et al., 2001). Wong et al. indicated that it was 1.5 times higher of the midazolam clearance in the patients with CYP3A5^*^1/^*^3 variant than that in patients with CYP3A5^*^3/^*^3 variant (Wong et al., 2004).

*Lorazepam*. Lorazepam is a benzodiazepine derivative with antianxiety and sedative-hypnotic properties. Lorazepam is mainly cleared by direct glucuronidation (Ameer and Greenblatt, 1981). UGTs primarily facilitate glucuronidation. The major drug substrate for UGT2B15 is lorazepam. Healthy individuals with the UGT2B15^*^2/^*^2 genotype had 0.58-fold lower clearance of lorazepam in comparison to UGT2B15^*^1/^*^1 genotype carriers, which suggested that the UGT2B15^*^2 variant might be an important determinant of interindividual PK (Chung et al., 2005).

*Diazepam*. Diazepam is another sedative of the benzodiazepine family that may be used in the ICU for sedation or anticonvulsant treatment. CYP 3A4 and 2C19 metabolize diazepam into its active metabolites, most importantly desmethyldiazepam (Calcaterra and Barrow, 2014). The marked interindividual variability in diazepam clearance is thought to be partially attributable to *CYP2C19* genetic variation. Bertilsson et al. indicated that compared with CYP2C19 PMs, NMs demonstrated higher clearance and shorter elimination half-life (t_1/2_) of diazepam (Bertilsson et al., 1989). Qin et al. pointed out that the elimination time of diazepam and desmethyldiazepam was prolonged in healthy Chinese subjects with mutated *CYP2C19* alleles (Qin et al., 1999). FDA approved label of diazepam states that, “the marked interindividual variability in clearance of diazepam is probably attributable to variability of *CYP2C19* and *CYP3A4*” (U.S Food and Drug Administration, 2018b).

*Dexmedetomidine*. Dexmedetomidine (DEX), used in the ICU to obtain light sedation and analgesia, is an effective α-2 adrenoreceptor agonist. There is marked variability in the efficiency of DEX to get the goal of light sedation, which has led investigators consider genetic variants. The metabolism of DEX is mainly related to the following genes, *CYP2A6*, α-2 adrenoreceptor genes (*ADRA2A, ADRA2B, ADRA2C*) and UGT genes (*UGT1A4, UGT2B10*) (Holliday et al., 2014). *CYP2A6* genetic variation did not affect the DEX clearance significantly in ICU patients (Kohli et al., 2012). The *ADRA2A* C1291G variant was associated with the prolonged time to take effect and the reduced sedation of individuals treated with DEX (Yagar et al., 2011). Although genetic variations may affect patients' response to DEX, additional investigation is required.

#### Antipsychotics

##### Haloperidol

Delirium affects up to 80% of patients in ICU and is a risk for death within the next year. Antipsychotics, particularly haloperidol, are the most commonly used drugs for delirium (Young et al., 2010). Haloperidol is metabolized partially by CYP2D6 to the active metabolite reduced haloperidol (Llerena et al., 1992). The DPWG has made recommendations for therapeutic doses of haloperidol basing on *CYP2D6* genotype. For CYP2D6 PMs, it is suggested to “reduce the therapeutic dose to 50% for haloperidol or select a substitute for haloperidol, such as fluphenazine, pimozide, quetiapine, flupenthixol, clozapine, or olanzapine” (Swen et al., 2011).

#### Antifungal Drugs

##### Voriconazole

Voriconazole is one of the azole antifungal drugs in the treatment of invasive aspergillosis and other severe fungal infections (Patterson et al., 2016). With wide variability among patients in systemic exposure and a narrow therapeutic range, it is not surprising that patient response is unpredictable and ADRs are common (Andes et al., 2009). Voriconazole concentrations have been associated with drug response and ADRs (e.g., hepatotoxicity, visual disturbances and visual hallucinations) (Pascual et al., 2008). The metabolism enzyme of voriconazole is CYP2C19, which is the principal pathway for elimination (Scholz et al., 2009). The doses of voriconazole needed to achieve targeted plasma concentrations increased in patients with the CYP2C19^*^17 variant which conveys an UM phenotype in comparison with CYP2C19^*^1/^*^1 carriers (NMs) (Weigel et al., 2015). Whereas, individuals carrying one or more CYP2C19^*^2 or ^*^3 variants are IMs or PMs, respectively and may have higher plasma concentrations. DPWG guideline recommends to “monitor serum concentrations for patients carrying the CYP2C19 PM or IM phenotype” (Swen et al., 2011). Evidence for an association between PM phenotype and ADRs with voriconazole was demonstrated in a case report recently (Lemaitre et al., 2013). An alternative agent is recommended to these patients because of the ADRs resulted by the increased voriconazole concentrations. Basing on this evidence, the CPIC guideline for voriconazole states that, “selecting an alternative agent that is not dependent on the metabolism of CYP2C19 in adults with CYP2C19 UMs, RMs or PMs” (Figure 1B) (Moriyama et al., 2016).

#### Cardiovascular Drugs

##### Anticoagulant drugs

*Warfarin*. As an oral anticoagulant used worldwide, warfarin is used to prevent and treat thromboembolic disorders in the ICU. The therapeutic index is narrow and interindividual variability in dose requirements is wide. The length of time to identify the ideal personalized dose can be as long as weeks to months resulting in the increased risk of thromboembolism or bleeding for patients. So warfarin dosing is a proverbial challenge (Kearon et al., 2016). The genes with the strongest literature support in affecting warfarin PK are *CYP2C9, CYP4F2*, and *VKORC1*. These genes when combined with clinical factors explain around 50% of dose variability.

Warfarin is a racemic mixture of the R and S stereoisomers and S-warfarin has 3–5 times more effect on inhabitation of VKORC1 than R-warfarin (Choonara et al., 1986). The predominant metabolic pathway of S-warfarin is via the CYP2C9 enzyme. Patients who carry more than one variant *CYP2C9* alleles have reduced S-warfarin clearance. Studies *in vitro* and *in vivo* showed the metabolism of S-warfarin could be impaired by the presence of CYP2C9^*^2 by the range of from 30% to 40% and ^*^3 alleles by the range of from 80 to 90% (Lee et al., 2002; Gage et al., 2017). There was a greater risk for patients carrying 1 or 2 copies of CYP2C9^*^2 or ^*^3 (PMs) to bleed during warfarin therapy in comparison with patients carrying homozygous CYP2C9^*^1 alleles (NMs). The required doses to achieve similar anticoagulation levels for patients carrying 1 or 2 copies of CYP2C9^*^2 or ^*^3 (PMs) were lower and the required time for them to meet a stable international normalized ratio (INR) was longer (Pirmohamed et al., 2013).

Warfarin anticoagulation is mediated through inhibition of VKORC1 which results in lower amounts of decreased vitamin K and reduced active clotting factors. As a rate-limiting enzyme, VKORC1 directly influences the efficiency of warfarin. Individuals who carrying one or two−1639A alleles of the *VKORC1* gene required lower doses of warfarin than those with−1639G/G alleles (Limdi et al., 2010).

The amount of reduced vitamin K and active clotting factors are decreased due to the transform of reduced vitamin K to hydroxyl-vitamin K1 by CYP4F2 enzyme. The CYP4F2^*^3 variant (rs2108622; c.1297G>A) was associated with reduced function resulting in approximate 10% increased warfarin dosage in A allele carriers (Gage et al., 2017).

The initial dosing of warfarin varies widely among individuals and it is affected by several clinical factors, including but not limited to race, age, gender, body weight, concomitant medications (e.g., factor Xa inhibitors), comorbidities, diet, as well as genetic factors. In general, the biggest proportion of known dose variability can be explained by *VKORC1* and *CYP2C9* gene variants. According to the 2017 update of CPIC guideline in regard to the application of PGx tests in dosing of warfarin, it takes more time (>2–4 weeks) for patients who carry reduced function genotypes such as *CYP2C9* ^*^1/^*^3, ^*^2/^*^2, ^*^2/^*^3, and ^*^3/^*^3 to obtain maximum INR than patients without these variants. The *VKORC1* variant (c.-1639G>A) profoundly influences the sensitivity of warfarin thereby carriers have lower dose requirements. The *CYP4F2* variant is also included in the dosing calculator and has been shown to improve dose predictions (Sun et al., 2016).

##### Antiplatelet drugs

*Clopidogrel*. Antiplatelet agent clopidogrel is commonly prescribed for acute coronary syndromes. It is an oral prodrug, activated via hepatic biotransformation to the active metabolite, which prohibits platelet activation and aggregation in a selective and irreversible way. Two oxidative steps involving CYP enzymes are required to form the active metabolite. CYP2C19 is the predominant enzyme. Individuals carrying CYP2C19^*^2 or ^*^3 alleles (PMs) have reduced active clopidogrel metabolite formation and greater platelet aggregation in comparison with CYP2C19^*^1 homozygotes (NMs) (Hulot et al., 2011). These low function alleles increase the risk for poor clopidogrel response and the ADRs (Cavallari et al., 2018). The FDA approved clopidogrel black box warning indicates the decreased effectiveness in patients carrying PM alleles (U.S Food and Drug Administration, 2018c). If no contraindication exists, alternative antiplatelet medications (e.g., prasugrel) are recommended to CYP2C19 PMs/IMs by the CPIC dosing guideline for clopidogrel. Individuals homozygous or heterozygous for CYP2C19^*^17 (UMs) have increased active clopidogrel metabolite formation and decreased platelet aggregation. Dosage of clopidogrel for CYP2C19 UMs is recommended by the CPIC guideline (Figure 1C) (Scott et al., 2013). DPWG guideline recommends “an alternative drug for the CYP2C19 PM and IM phenotypes” (Swen et al., 2011). Clopidogrel also undergoes extensive hydrolytic metabolism to the inactive metabolite by hepatic carboxylesterase 1 (CES1) (Tang et al., 2006). *CES1* genetic variation may result in the deficient CES1 hydrolysis activity. The hydrolysis of clopidogrel was marked impaired in patients with the D260fs and G143E *CES1* genetic variants (Zhu et al., 2013). Hence, it may be associated with the increased plasma concentrations of active metabolite of clopidogrel.

##### Antiarrhythmic drugs

*Metoprolol*. Metoprolol is a beta-blocker often used as an antiarrhythmic. It undergoes α-hydroxylation and *O*-demethylation as a substrate of CYP2D6 enzyme. *CYP2D6* genetic variants have a significant impact on metoprolol PK. CYP2D6 PMs had significantly higher metoprolol plasma concentrations and a prolonged half-life (t_1/2_) compared to other CYP2D6 phenotypes, despite the same doses of metoprolol administered to PMs and non-PMs (Rau et al., 2009). CYP2D6 PMs also appeared to have increased risk of metoprolol toxicity (Isbister et al., 2016). The DPWG guideline recommends “a reduced dose of metoprolol by 70–75% or an alternative agent, such as bisoprolol or carvedilol to PMs with heart failure.” In PMs with other indications, the DPWG guideline suggests to “remain alert to ADRs, such as cold extremities and bradycardia, or choose alternative drugs, such as bisoprolol and atenolol.” In IMs with heart failure, “a 50% reduction of metoprolol dose or selection of alternative drugs, such as bisoprolol and carvedilol,” is recommended. While in IMs with other indications, the guideline recommends to “remain alert to ADRs or select alternative drugs” (Rau et al., 2009). UMs are recommended to “an alternative agent (e.g., bisoprolol or carvedilol for heart failure, atenolol or bisoprolol for other indications) or titration of the metoprolol dose to 1.5 times higher than the normal dose needed for efficacy” (Goryachkina et al., 2008; Swen et al., 2011).

*Carvedilol*. Carvedilol is another commonly prescribed beta-blocker in cardiovascular medicine. CYP2D6 is carvedilol's primary metabolizing enzyme. *CYP2D6* alleles and carvedilol PK are significantly associated with each other. In healthy volunteers, however, there was no association between the *CYP2D6* genotypes and cardiovascular indicators (e.g., blood pressure, heart rate). In the same study, *ADRB1* Gly49 was found to be associated with increased carvedilol effects on exercise heart rates (Sehrt et al., 2011). The DPWG guideline recommends that no action is needed for carvedilol and *CYP2D6* genotype. For CYP2D6 PMs, DPWG states that, “the plasma concentration of carvedilol can be elevated, but this does not result in an increase of ADRs” (Swen et al., 2011). The FDA-approved drug labeling for carvedilol states that, “plasma concentrations of carvedilol may be higher in CYP2D6 PMs compared to NMs,” but does not discuss altering carvedilol dosing based on a patient's *CYP2D6* genotype (U.S Food and Drug Administration, 2017a). No CPIC guideline is available for recommendations.

*Propafenone*. Propafenone is an antiarrythmic used for the prevention of the recurrence of atrial fibrillation in individuals with episodic atrial fibrillation and no underlying structural heart disease and in the management of paroxysmal supraventricular tachycardia and atrial flutter. The metabolizing enzymes for propafenone are CYP3A4, CYP2D6, and CYP1A2 enzymes. Multiple studies have showed that genetic variants of *CYP2D6* gene influence the plasma concentrations of propafenone. Standard doses of propafenone in CYP2D6 PMs may lead to higher plasma parent drug concentrations in comparison with NMs. The drug label approved by FDA for propafenone recommends the dosing regimen is the same for all patients (CYP2D6 PMs and NMs) (Rouini and Afshar, 2017). The DPWG guidelines recommend, “CYP2D6 PMs receive a 70% reduction in initial dose, plus electrocardiogram and plasma concentration monitoring.” For CYP2D6 IMs and UMs, the guideline states that data which calculates the dose adjustment is insufficient and recommends that, “the drug dose adjustment should be based on plasma concentration and the electrocardiogram, or it is encouraged to choose alternative drugs, such as sotalol, quinidine, disopyramide, or amiodarone” (Mörike et al., 2008).

##### Vasoactive drugs

*Vasopressin*. Vasopressin is recommended to treat septic shock. More than 10% of patients treated with vasopressors develop life-threatening ADRs, including myocardial infarction, limb and mesenteric ischemia. Anantasit et al. indicated that there were close associations between serious ADRs and a variant near the *AVPR1b* gene, the AA allele at rs28418396 (Anantasit et al., 2014). No prescribing recommendations based on PGx are available to date.

##### Statins

*Simvastatin*. Simvastatin is one of the HMG-CoA reductase inhibitors used to reduce cholesterol. Skeletal muscle toxicity is a rare but a serious statin-related ADR occurring in 1–5% of subjects. OATP1B1, encoded by *SLCO1B1* gene, is a hepatic transporter which facilitates statin uptake by the liver (Couvert et al., 2008). *SLCO1B1* variants significantly altered the PK of simvastatin (Niemi, 2010). SLCO1B1^*^5, ^*^15, and ^*^17 genotypes were associated with lower level of plasma clearance of simvastatin. A single coding variant in *SLCO1B1*, rs4149056T>C, was associated with the higher plasma concentration of simvastatin, and hence the risk of ADRs. Pasanen et al. pointed out that compared to the patients with TT homozygous at rs4149056 in *SLCO1B1*, the active simvastatin was significantly higher in those with CC homozygous (Pasanen et al., 2006). Nearly 1/4 of the general population is a carrier of C genotype. The variant has also been associated with lower simvastatin adherence presumably due to the development of muscle toxicity (Voora et al., 2009). In the Study of the Effectiveness of Additional Reductions in Cholesterol and Homocysteine (SEARCH), the association between rs4149056 SNP and myopathy was observed. This study also showed an association between this SNP and the cholesterol-lowering effects of simvastatin. Another noncoding SNP, rs4363657, which is in almost complete linkage disequilibrium with rs4149056, was also found to be strongly associated with statin-induced myopathy (Group et al., 2008). The simvastatin CPIC guideline recommends, “the *SLCO1B1* genotype be used to identify individuals at greater risk for simvastatin related myopathy while receiving a lower dose of simvastatin (e.g., 40 mg daily).” In individuals heterozygous or homozygous for the C allele, the guideline recommends, “to use lower simvastatin doses or consider alternative statins like pravastatin and rosuvastatin” (Ramsey et al., 2014). A simvastatin drug-drug interaction occurring in individuals with this variant may enhance the risk for myopathy.

#### Gastroenterological Drugs

##### Proton pump inhibitors

Proton pump inhibitors (PPIs) are frequently used to patients in the ICU to prevent stress ulcers and treating gastrointestinal bleeding. Up to 20% of patients who received PPIs orally could not achieve ideal acid suppression (Shimatani et al., 2006). More than 80% of the PPIs (e.g., pantoprazole, lansoprazole, omeprazole, esomeprazole, but not rabeprazole) are metabolized by CYP2C19 and CYP3A4 enzyme (Furuta et al., 2005). Genetic variations of the CYP2C19 enzyme may result in differences among PPIs in response. In CYP2C19 NMs treated with PPIs orally, the plasma concentration of PPIs was decreased compared to PMs (Chaudhry et al., 2008). The clearance of lansoprazole, omeprazole, and pantoprazole were higher in CYP2C19 RMs compared to PMs, resulting in decreased plasma concentration of parent (Shin and Kim, 2013). *CYP2C19* RM genotype was found the most relevant PGx factor in clinical practice (Shimatani et al., 2006). The DPWG recommends for those with the CYP2C19 UM phenotype, “extra caution during treatment due to a higher risk of insufficient response to omeprazole, esomeprazole, lansoprazole, and pantoprazole.” Further recommendation is to increase the doses by 200, 50–100, 100–200, and 400%, respectively (Swen et al., 2011). The CPIC guideline for CYP2C19 and PPIs (e.g., esomeprazole, omeprazole, etc.) is in progress.

##### Ondansetron

Ondansetron is prescribed to prevent vomiting and postoperative nausea in the ICU (Apfelbaum et al., 2013). CYP2D6 is the primary metabolizing enzyme of ondansetron (Sanwald et al., 1996). There is a link between the variability in efficacy of ondansetron and the *CYP2D6* genotype. The evidence was observed that antiemetic effect of ondansetron was decreased in CYP2D6 UMs treated for chemotherapy-induced and postoperative nausea or vomiting (Stamer et al., 2011). According to the CPIC guideline for ondansetron, if the genotype of *CYP2D6* is known, “Alternative 5-HT3 receptor antagonist antiemetics not metabolized by CYP2D6 (e.g., granisetron) should be considered in CYP2D6 UMs” (Bell et al., 2017). Compared with CYP2D6 NMs, the incidence of vomiting in IMs and PMs were the same (Kaiser et al., 2002). There is no dose reduction recommended in FDA labeling. Besides *CYP2D6* genetic variants, the *ABCB1* gene was implicated in affecting the response to ondansetron (Lehmann et al., 2013; He et al., 2014). 5-HT3 receptor antagonist, such as ondansetron, could prolong the QT interval. There is a fatal risk of polymorphic ventricular tachycardia in the patients with the syndrome of congenital prolongation of the QT interval of the electrocardiogram. Therefore, potentially increased blood levels of ondansetron in CYP2D6 IMs or PMs might be at an even greater risk for QT interval prolongation (Zuo et al., 2014). In this case, ondansetron should not be used in these patients.

#### Anticonvulsant Drugs

##### Phenytoin

Phenytoin is an anticonvulsant medication that is used for both focal and generalized status epilepticus. It has a narrow therapeutic index, nonlinear PK and large variability among patients. Phenytoin has several dose and concentration-related ADRs (e.g., neurologic, hypotension and arrhythmias) and initial maintenance dose selection is important. One enzyme involved in the metabolism of phenytoin is CYP2C9, and variant *CYP2C9* alleles can impact the concentrations of phenytoin. Clearance rates of phenytoin in individuals with reduced activity *CYP2C9* variants may be reduced. Meanwhile, patients with this variant will face a greater risk for dose related side effects (Caudle et al., 2014b). Phenytoin maintenance doses were lower in heterozygous individuals who carried 1 reduced activity allele and lower by 31–52% for individuals who carried 2 reduced activity *CYP2C9* alleles compared to CYP2C9^*^1/^*^1 (NM) (van der Weide et al., 2001). To avoid excessive blood concentrations, a decrease of 25% in the starting maintenance dose for CYP2C9 IMs (CYP2C9^*^1/^*^3 and CYP2C9^*^1/^*^2 genotypes) is recommended in the CPIC guideline. For CYP2C9 PMs (CYP2C9^*^2/^*^2, CYP2C9^*^2/^*^3, CYP2C9^*^3/^*^3), a decrease of 50% of starting maintenance dose should be considered (Caudle et al., 2014b). DPWG guideline has the same recommendations with the CPIC guideline that, “to reduce the starting maintenance dose of phenytoin basing on *CYP2C9* genotype and monitor serum concentrations and observe closely for ADRs” (Swen et al., 2011).

Variants in the *HLA-B* gene are exposed to a greater risk of serious phenytoin-induced cutaneous ADRs: toxic epidermal necrolysis (TEN) and Stevens-Johnson syndrome (SJS). The rates of death for SJS and TEN are approximately 5% and 30% respectively; sepsis related to loss of skin integrity is considered to be the critical cause of death (Roujeau and Stern, 1994). The incidence of phenytoin induced SJS and TEN was high in HLA-B^*^15:02 allele carriers. HLA-B^*^15:02 allele with high frequency in Han Chinese and other Asians can increase the chance of suffering from SJS/TEN (Hung et al., 2010). HLA-B^*^15:02 allele is distinct in race and regional distribution. Southeast Asia population has a frequency of HLA-B^*^15:02 allele as high as >15% (Chung et al., 2004). It is estimated that the frequency of HLA-B^*^15:02 allele in Yunnan province of China is as high as 36%. There is high risk for these serious cutaneous ADRs in individuals carrying one or two HLA-B^*^15:02 alleles. The guideline recommends “using an alternative anticonvulsant (not carbamazepine) only when the advantages of treating the disease obviously outweigh the disadvantages” (Figure 1D) (Caudle et al., 2014b). The FDA also makes recommendations that individuals who carrying HLA-B^*^15:02 allele should avoid to use phenytoin (U.S Food and Drug Administration, 2017b).

##### Carbamazepine

Carbamazepine is the commonly used antiepileptic drugs in the treatment of generalized and tonic-clonic seizures as well as neuropathic pain. Similar to phenytoin, the most severe manifestations of carbamazepine induced cutaneous ADRs is SJS/TEN. The CPIC guideline and FDA recommend to screen the HLA-B^*^15:02 variant in the populations at risk before starting carbamazepine (Figure 1D) (Leckband et al., 2013). The risk in the Chinese population is 10 times greater than in Caucasians. It is not recommended for carbamazepine-naive individuals who possess one or two HLA-B^*^15:02 alleles to take carbamazepine. The frequency of the HLA-A^*^31:01 variant in Han Chinese populations, Northern European populations and Japanese populations is 2, 2–5, and 9%, respectively (Wen et al., 2008; Schmidt et al., 2009; Ozeki et al., 2011). McCormack et al. found that this allele was significantly associated with carbamazepine-induced hypersensitivity reactions among subjects of Northern European ancestry. The presence of the allele increased the risk from 5.0 to 26.0%, whereas its absence reduced the risk from 5.0 to 3.8% (McCormack et al., 2011).

##### Valproic acid

Valproic acid is an anticonvulsant drug used in status epilepticus and to treat epilepsy. Valproic acid is metabolized by CYP450 enzymes (CYP2A6, CYP2B6, CYP2C9, and CYP3A5) and also by the UDT enzymes (e.g., UGT1A6, UGT2B7, UGT2B15, etc.) (DeVane, 2003). Valproic acid has a narrow therapeutic range and high interindividual variability in PK and PD (Chadwick, 1985). PGx studies of valproic acid are comparatively few in comparison with that of carbamazepine and phenytoin. A study showed an association between *UGT1A6* variants and higher valproic acid maintenance doses as compared with children without variant alleles (Guo et al., 2012). *UGT2B7* is also involved in the glucuronidation of valproic acid. Chung et al. found that there was a noteworthy but nonsignificant growth in the AUC of valproate in the patients with increased UGT2B7^*^2 alleles (Chung et al., 2008). About 6–14% of interindividual variability in valproic acid PK can be explained by *CYP2B6* variant alleles, *CYP2A6* and *CYP2C9* genes. Children who carried the CYP2A6^*^4, CYP2B6^*^6, or CYP2C9^*^3 alleles was likely to obtain high concentrations of valproic acid (Jiang and Wang, 2004).

### PGx in the Context of Drug-Drug Interactions

Most ICU patients have multiple medical problems and require treatments with numerous medications, often new to their therapeutic regimens. Five main CYP enzymes (CYP2D6, CYP2C9, CYP2C19, CYP3A4, and CYP1A2) are involved in the metabolism of over 90% of therapeutic drugs (Rendic and Guengerich, 2015). If CYP isoforms is inhibited or induced by one drug, the metabolic status of other drugs will likely be changed *in vivo*, which can result in decreased therapeutic efficacy or acute drug toxicity (Doucet et al., 2002). Thus, the potential risks of polypharmacy related ADRs resulted from drug-drug interaction are critical in ICU patients (Empey, 2010). For example, voriconazole is an effective inhibitor of CYP enzymes (e.g., CYP2B6, CYP2C9, CYP2C19, and CYP3A) and meanwhile is metabolized by several CYP enzymes (Brüggemann et al., 2009). Combination use of high dose phenytoin or carbamazepine (CYP enzyme inducers) with voriconazole significantly decreased mean AUC and Cmax of voriconazole, potentially reducing its effectiveness (Li et al., 2017). On the other hand, CYP2C19 inhibitors (e.g., cimetidine and omeprazole) may result in greater increases in voriconazole concentrations in patients (Bouatou et al., 2014; Qi et al., 2017). Further, CYP interaction of voriconazole with midazolam caused decreased clearance of midazolam (Saari et al., 2006). Another ICU drug-drug interaction example is between PPIs and clopidogrel, which are commonly co-prescribed in order to minimize antiplatelet treatment-related gastrointestinal bleeding. Some of these PPIs, such as omeprazole, are inhibitors of CYP2C19, so use of PPIs may affect clopidogrel response (Scott et al., 2013). Both concomitant medication of clopidogrel with a high omeprazole dose (80 mg) and taking clopidogrel and omeprazole separated lead to decreases of the plasma concentrations of the active metabolite of clopidogrel compared to clopidogrel administered alone (Angiolillo et al., 2011). These results suggested the metabolic drug-drug interaction between PPIs and clopidogrel.

In addition to drug-drug interaction, our review shows that the effect of a drug can be affected by PGx as well. Therefore, to interpret and subsequently predict a drug effect in the ICU, one needs to consider drug-drug interaction and drug-gene interaction (PGx). For example, metoprolol is metabolized predominantly by CYP2D6, a highly polymorphic gene. Meantime, CYP2D6 can be inhibited by a number of drugs, such as quinidine, fluoxetine, paroxetine, and H1-receptor antagonists. Therefore, the FDA approved drug labeling for metoprolol states that, “CYP2D6 PMs, and EMs who concomitantly use CYP2D6 inhibiting drugs will have increased metoprolol blood levels” (U.S Food and Drug Administration, 2018d). Here, the drug label includes both the genetic effect in CYP2D6 PMs and the drug-drug interaction effect in CYP2D6 EMs. Diphenhydramine is a widely used classic H1-receptor antagonist that has been shown to inhibit the CYP2D6 *in vitro* (Hamelin et al., 1998). Indeed, diphenhydramine coadministration significantly increased the peak concentration of metoprolol in CYP2D6 EMs (Hamelin et al., 2000). This is just an example where enhanced understanding of PGx in the context of drug-drug interactions can help optimize the dosing of these drugs as well as enable dosage adjustments to prevent toxicities with other drugs.

## Discussion

### Application of PGx in the ICU

PGx is an evolving field that provides the promise of personalized selection of medication and optimized dosage to maximize therapeutic response and to minimize ADRs. In the ICU, where insufficient drug efficacy, polypharmacy and ADRs are challenges faced by clinicians, PGx guided prescribing may be of immense value. Specific areas to consider include:

*Personalized Dosing:* Studying the interplay between PGx, population PK and clinical PK might most effectively advance PGx for personalized dosing. The warfarin dose calculator at the *warfarin dosing.org* website is commonly used for prediction of warfarin doses and uses genotypes and clinical factors in its estimates (Johnson et al., 2017). This approach should be evaluated for other medications used in the ICU as we gain scientific information on PGx.

*Decrease ADRs:* Many factors are involved in the variation of the efficacy or ADRs to pharmacotherapy. The effect of genetics on drug PK/PD is increasingly being appreciated. PGx provides clinicians in the ICU with additional information to assess the potential for efficacy and/or toxicity of medications. For instance, there was a high risk of the phenytoin induced SJS or TEN in individuals who carry one or two HLA-B^*^15:02 alleles (van der Weide et al., 2001). The clinical implementation of PGx knowledge is expected to decrease ADRs by improving the selection of medicines who are likely to be safe and selecting the optimum dose for efficacy in critically ill patients (Empey, 2010).

*Save costs and time to optimize therapy:* The goal of PGx is to facilitate to select the right medication and the tailored dosage. The implementation of PGx in the ICU has the potential to significantly reduce the costs and time. According to Desta et al., the *H. pylori* infection eradication rate of the tailored treatment basing on PPIs PGx was higher and the final cost per patient for successful eradication did not increase (Desta et al., 2002). These estimates indicate that the determination of *CYP2C19* genotype prior to PPI therapy is cost-effective. In 12 large academic institutions in the U.S., genotype-guided antiplatelet treatments were found to decrease the incidence of cardiovascular events and increase the cost-effectiveness in patients who underwent percutaneous coronary interventions (Deiman et al., 2016). Different genotyping/phenotyping analysis, like those for *CYP2D6*, have already been successfully applied in oncology practice and are becoming increasingly indicated in psychiatric practice. This is particularly true when variants in one gene were shown to be important in the PK and response to multiple drugs. Several PGx guided treatments have been demonstrated to have high cost-effectiveness in chronic disease settings (Empey et al., 2017). Given that some of these drugs are commonly used in the ICU (e.g., *CYP2C19* genotype for voriconazole, clopidogrel, PPIs, diazepam treatment), it is possible that the inclusion of PGx information for patients in the ICU could also be cost-effective. The cost-effectiveness of genotyping in guiding choice of multiple medications for critically ill patients requires further scientific study.

Despite all the promises, PGx has not been studied or implemented extensively in the ICU setting to date, due in part to the hurdles presented. Results of diagnostic tests to identify genotypes would need to be available quickly and clinicians would need to understand how to interpret and apply the results in order to guide therapy efficiently. Secondly, PK/PD parameters can be affected by the changing blood volume, severity of illness, and not only acute but also chronic diseases impacting organ function. To date, most PGx studies were conducted in healthy volunteers with single specific disease states, not critically ill patients. It is unlikely accurate to forecast or monitor drug response basing on the PGx knowledge generated in healthy individuals or individuals with chronic illness. Thirdly, it is common in the ICU for patients to be treated with more than 5 drugs. The interpretation of PGx in the ICU will be further complicated by the existence of polypharmacy and numerous potential drug-drug interactions. Fourth, due to our limited knowledge of the mechanisms of ADRs, assessing how many ADRs are associated with genetic variations is still unknown. And finally, the PGx study end points assessed were often related to PK, which may or may not reflect the clinical response/toxicity of patients.

### Future Perspective

With the improved accuracy and rapidly declining costs of large-scale genome testing and development of cost-effective array-based tests, technical advancement has gradually overcome the first challenge (Johnson et al., 2012; Schildcrout et al., 2012). Indeed, it is increasingly easy, cheap and straightforward to perform PGx tests in clinical practice (particularly those around metabolism gene and drug transporters) considering the commercialization and routine utility of PGx tests. In spite of the recent increase in the knowledge of PGx used for targeted therapeutics, acutely ill patients in the critical care environment fail to be routinely evaluated for genetic variants to forecast the efficacy of the treatment. The CPIC/DPWG guidelines and FDA labeling clearly support using data when it is available. To date, the number of PGx studies done in the ICU setting remains small. More research is needed in this particularly challenging area to gather the evidence and justify widespread PGx screening in the ICU. It is challenging for critical care researchers to design and conduct studies on gene association that are adequately controlled but also possess the power to correctly assess findings and apply the PGx information clinically. The proper time point to order the PGx tests or whether it should be done in all patients is rarely discussed. Given the complexity and the hurdles in studying and implementing PGx in the ICU setting, we propose the following critical steps for practitioners. First of all, perform preemptive genotyping and construct clinical recommendation tool in the electronic medical record. This will allow the timely and accurate application of genotype information, which can be used for prescribing decisions at the point-of-action. Secondly, focus on key gene products and evaluate them as a panel. The evidence to date (as reviewed in this manuscript) supports the genotyping of key metabolic enzymes to guide pharmacotherapy of commonly used ICU drugs. The predictive value of a single genetic variant with regard to drug response is often limited, and combinations of multiple genetic variants may be involved. Thirdly, develop clinically actionable flow charts (Figure 1) to facility the adaptation of genomic information into clinical decision-making. In the ICU, a clinically actionable flow chart should consider both gene-drug interactions and drug-drug interactions when recommending medications to treat an ICU patient. And finally, offer basic and advanced PGx education to all healthcare professionals caring for ICU patients so the interprofessional team understands the clinical application of PGx and how it can further improve patient outcomes. In summary, PGx is moving forward from the bench to the bedside and will offer new insight to assist clinicians in optimizing pharmacotherapy in the ICU. Further research is needed to define cost-effective, clinically actionable recommendations in the ICU, but the interest and potential of precision medicine to improve pharmacotherapy is compelling.

## Author Contributions

SZ and RH conceived and designed the study. SZ conducted the literature search and drafted the manuscript; SZ, DS, PJ, and RH edited and approved the manuscript.

### Conflict of Interest Statement

The authors declare that the research was conducted in the absence of any commercial or financial relationships that could be construed as a potential conflict of interest.

## Abbreviations

ICU: intensive care unit
PGx: pharmacogenomics
ADR: adverse drug reaction
FDA: food and drug administration
CPIC: clinical pharmacogenetics implementation consortium
DPWG: royal dutch association for the advancement of pharmacy-pharmacogenetics working group
CYP: cytochrome P450
UM: ultrarapid metabolizer
RM: rapid metabolizer
EM: extensive metabolizer
NM: normal metabolizer
IM: Intermediate metabolizer
PM: poor metabolizer
PK: pharmacokinetics
PD: pharmacodynamics
M3G: morphine-3-glucuronide
M6G: morphine-6-glucuronide
UGT: uridine diphosphate glucuronosyltransferase
AUC: area under the curve
DEX: Dexmedetomidine
INR: international normalized ratio
PPIs: proton pump inhibitors
TEN: toxic epidermal necrolysis
SJS: stevens-johnson syndrome.
